# Prevalence and Predictors of Pre-Diabetes and Diabetes among Adults 18 Years or Older in Florida: A Multinomial Logistic Modeling Approach

**DOI:** 10.1371/journal.pone.0145781

**Published:** 2015-12-29

**Authors:** Ifechukwude Obiamaka Okwechime, Shamarial Roberson, Agricola Odoi

**Affiliations:** 1 Department of Biomedical and Diagnostic Sciences, College of Veterinary Medicine, University of Tennessee, Knoxville, Tennessee, United States of America; 2 Florida Department of Health, Bureau of Chronic Disease Prevention, Tallahassee, Florida, United States of America; National Institute of Health, ITALY

## Abstract

**Background:**

Individuals with pre-diabetes and diabetes have increased risks of developing macro-vascular complications including heart disease and stroke; which are the leading causes of death globally. The objective of this study was to estimate the prevalence of pre-diabetes and diabetes, and to investigate their predictors among adults ≥18 years in Florida.

**Methods:**

Data covering the time period January-December 2013, were obtained from Florida’s Behavioral Risk Factor Surveillance System (BRFSS). Survey design of the study was declared using SVYSET statement of STATA 13.1. Descriptive analyses were performed to estimate the prevalence of pre-diabetes and diabetes. Predictors of pre-diabetes and diabetes were investigated using multinomial logistic regression model. Model goodness-of-fit was evaluated using both the multinomial goodness-of-fit test proposed by Fagerland, Hosmer, and Bofin, as well as, the Hosmer-Lemeshow’s goodness of fit test.

**Results:**

There were approximately 2,983 (7.3%) and 5,189 (12.1%) adults in Florida diagnosed with pre-diabetes and diabetes, respectively. Over half of the study respondents were white, married and over the age of 45 years while 36.4% reported being physically inactive, overweight (36.4%) or obese (26.4%), hypertensive (34.6%), hypercholesteremic (40.3%), and 26% were arthritic. Based on the final multivariable multinomial model, only being overweight (Relative Risk Ratio [RRR] = 1.85, 95% Confidence Interval [95% CI] = 1.41, 2.42), obese (RRR = 3.41, 95% CI = 2.61, 4.45), hypertensive (RRR = 1.69, 95% CI = 1.33, 2.15), hypercholesterolemic (RRR = 1.94, 95% CI = 1.55, 2.43), and arthritic (RRR = 1.24, 95% CI = 1.00, 1.55) had significant associations with pre-diabetes. However, more predictors had significant associations with diabetes and the strengths of associations tended to be higher than for the association with pre-diabetes. For instance, the relative risk ratios for the association between diabetes and being overweight (RRR = 2.00, 95% CI = 1.55, 2.57), or obese (RRR = 4.04, 95% CI = 3.22, 5.07), hypertensive (RRR = 2.66, 95% CI = 2.08, 3.41), hypercholesterolemic (RRR = 1.98, 95% CI = 1.61, 2.45) and arthritic (RRR = 1.28, 95% CI = 1.04, 1.58) were all further away from the null than their associations with pre-diabetes. Moreover, a number of variables such as age, income level, sex, and level of physical activity had significant association with diabetes but not pre-diabetes. The risk of diabetes increased with increasing age, lower income, in males, and with physical inactivity. Insufficient physical activity had no significant association with the risk of diabetes or pre-diabetes.

**Conclusions:**

There is evidence of differences in the strength of association of the predictors across levels of diabetes status (pre-diabetes and diabetes) among adults ≥18 years in Florida. It is important to monitor populations at high risk for pre-diabetes and diabetes, so as to help guide health programming decisions and resource allocations to control the condition.

## Introduction

Diabetes is a metabolic disease characterized by high blood sugar or glucose levels, resulting from worsening or severe insulin resistance. It is observed in individuals with a glycated hemoglobin (also called hemoglobin A1c, HbA_1c_, A1C or Hb_1c_) level of 6.5% or higher, a Fasting Plasma Glucose (FPG) of 126 mg/dl or higher, and Oral Glucose Tolerance Test (OGTT) levels of 200 mg/dl or higher [1, 2]. However, the precise mechanisms that lead to diabetes remain unknown [3]. Pre-diabetes, also known as borderline diabetes or intermediate hyperglycemia, is observed in individuals with an A1c of 5.7% to 6.4%, an FPG level from 100 mg/dl to < 126 mg/dl and an OGTT > 200 mg/dl [4–6]. It results when either the pancreatic β-cells do not produce sufficient insulin to dispose off blood glucose, or the body does not use the insulin well enough in order to lower blood glucose levels, or as a result of failure of the pancreatic β-cells to secrete insulin. Pre-diabetes increases the risk of type 2 diabetes that consequently predisposes individuals to heart disease, stroke, nerve damage, kidney failure, and eye problems [5, 7]. Nevertheless, studies report that for pre-diabetic patients, lifestyle modification can help prevent or reduce its progression to diabetes by 40–70%, which emphasizes the need for early diagnosis [8, 9].

Globally, the prevalence of diabetes is on the rise with an estimated 387 million diabetics; and it is estimated that by 2035, 592 million people will have diabetes [8, 10]. Unfortunately, the actual global prevalence of pre-diabetes is unknown. Thus, despite the potential for a rise in the prevalence, and the serious complications associated with the disease, many public health planners and policy makers remain generally unaware of its current prevalence, and the variations in significant risk factors for both pre-diabetes and diabetes [11]. In the United States alone, at least 86 million adults aged ≥ 20 years old are currently pre-diabetic with 51% of adults aged 65 years or older having the disease. Moreover, 9 out of 10 people with pre-diabetes don’t know they have it; 1 in 3 American adults are living with pre-diabetes; 15–30% of individuals with pre-diabetes will develop type 2 diabetes and there are currently 29.1 million adults in America with diabetes [12].

Geographic disparities in the prevalence of the conditions are also known to exist with the Southeast region of the US, including Florida, having higher rates of diabetes and other chronic diseases, compared to other parts of the country. Moreover, Florida has the highest number (1.2 million) of people living with pre-diabetes and approximately 1.7 million diabetics [13]. The high risk of diabetes in the area are mainly due to modifiable (30%) and non-modifiable (37%) risk factors [14]. It is estimated that healthcare costs are 2.3 times higher in patients with diabetes than those without the condition; and there is a 50% higher risk of mortality among individuals with diabetes than those without the condition [15].

It is generally assumed that the risk factors for pre-diabetes are the same as those for diabetes. However, it is unclear if the strength of association of the risk factors are the same for both pre-diabetes and diabetes. Most previous studies have investigated risk factors for pre-diabetes and diabetes as binary variables in separate models. Thus, no studies have investigated the two conditions as a polytomous variable in the same model, while investigating the association of each level of the polytomous variable with suspected risk factors. Moreover, there is little data available on the current prevalence of pre-diabetes. Understanding the current burden of each of the conditions and their predictors is important for guiding programing decisions to combat the conditions. Thus, the objective of this study was to estimate the prevalence of pre-diabetes and diabetes and to investigate their predictors among adults ≥18 years in Florida. The findings of this study will be important for guiding programming decisions, resource allocation, and for informing public health policy decisions.

## Methods

### Study Area

The study area included all the 67 counties in the state of Florida. Florida was chosen because it is thought to have one of the highest number of people living with these conditions in the country [13]. Based on the 2010 population census, the state consisted of 4,245 census tracts and had an estimated total population of approximately 18.8 million people. The state has a mixture of urban and rural areas with Miami-Dade County being the most urban, and most populated with 2.5 million residents, and Liberty County being the most rural and least populated with 8,365 residents. About 75% of the population are white, 16% are black and 9% comprise of other races [16]. Ethnically, non-Hispanics make up 77.5% of the population while 22.5% comprise the remainder of the population. Approximately 49% are male, while 51% are female. The majority of the population are aged 18 years and older with the largest percentage (26%) being in the 35–49 years age category. Eight percent, 16%, 25%, and 22% of the adult population are aged 20–24, 25–34, 50–64, and ≥ 65 years respectively. Approximately 21% of the population are between 0 and 18 years.

### Data Source and Variable Selection

Data for this study, covering the time period January-December 2013, were obtained from Florida’s Behavioral Risk Factor Surveillance System (BRFSS), an on-going health telephone-based questionnaire survey conducted with technical and methodological support from the Centers for Disease Control and Prevention (CDC) [17]. Collection of the BRFSS data involved monthly telephone interviews of a random sample of all non-institutionalized civilian residents aged 18 years or older. To gather a representative sample, a multistage design based on Random Digit Dialing (RDD) was used to obtain both landline and cell phone numbers. The questionnaire was administered via telephone interviews by a survey company. However, the Florida Department of Health continually monitored the telephone interviews to ensure data quality. The collected data were sent to the CDC. The CDC collated and then sent the data to the Florida Department of Health. The interview questionnaire included questions on health-related risk behaviors, chronic health conditions, and the use of preventive services. Access to the de-identified data was granted by the Florida Department of Health.

**Fig 1:**
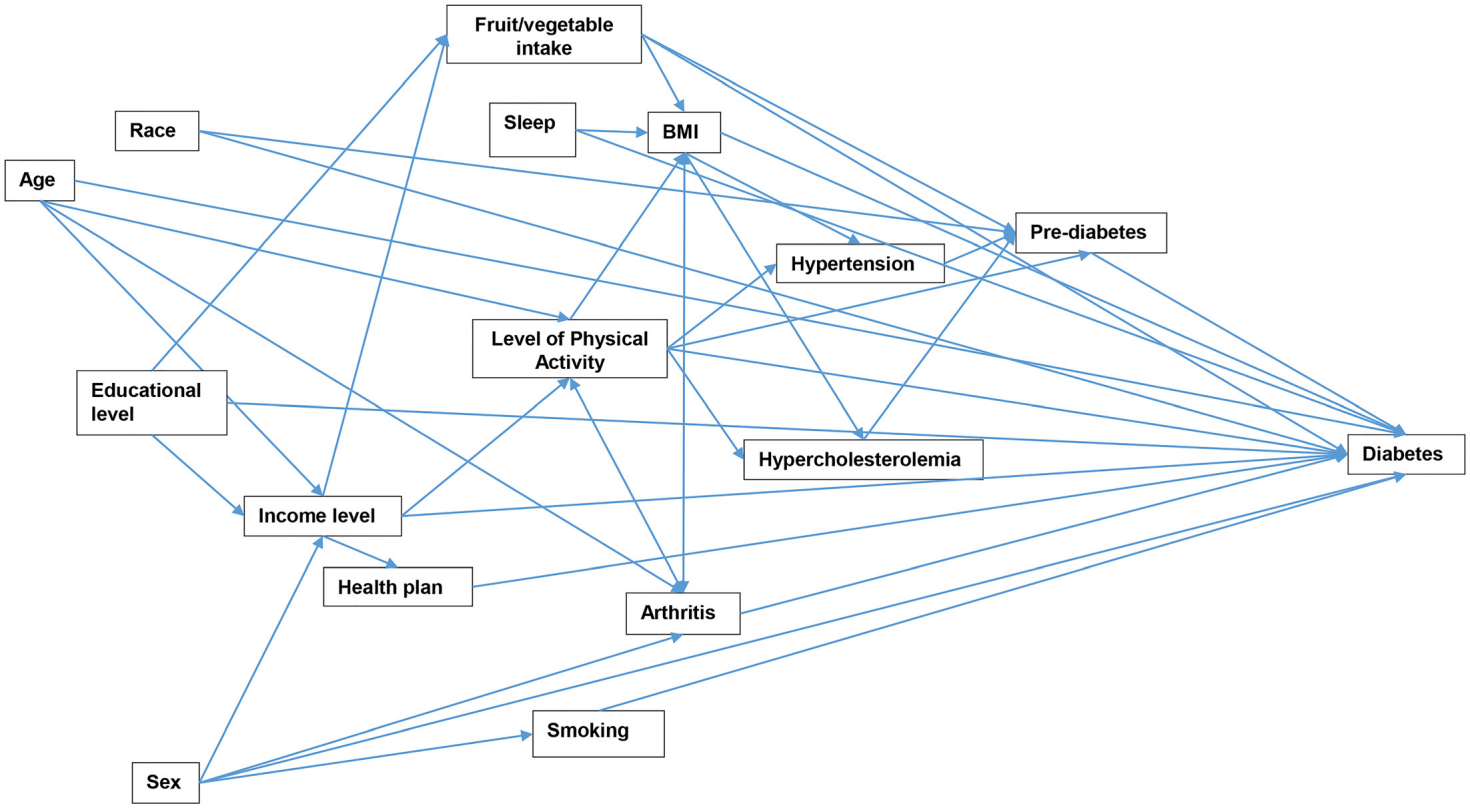
Conceptual model Representing Predictors of Pre-diabetes and Diabetes in Adults 18 Years and Older in Florida, 2013

The outcome variable of interest was diabetes status (Diabetes, pre-diabetes, and no diabetes or pre-diabetes). The questionnaire and codebook were reviewed in order to identify questions and variables that would be useful in identifying the diabetes status as well as potential predictors of diabetes or pre-diabetes. Diabetes status was defined on the basis of survey respondents having reported being informed by a physician that they had pre-diabetes or diabetes. However, pregnancy diabetes was not included in the definition and no distinction was made between type 1 and 2 diabetes.” Participants were identified as having pre-diabetes or diabetes, if they reported being told by a doctor that they had either conditions. Participants who reported being told by a doctor that they had diabetes, or did not have either diabetes or pre-diabetes were also included in the study. Based on a conceptual model (Fig 1), 16 potential predictors of diabetes status were considered for investigation: age, sex, race, marital status, body mass index (BMI), physical activity, quantity of sleep, smoking status, fruit intake, vegetable consumption, hypertension, high cholesterol, arthritis, education, income level and having health insurance.

### Data Preparation and Descriptive Analyses

A single diabetes status variable was created by combining responses to the pre-diabetes and diabetes questions. Respondents who reported being told by a doctor that they had pregnancy diabetes were excluded. Race/ethnicity was re-coded by combining non-Hispanic Asians, American Indian/Alaskan Natives and other race as one category; leaving non-Hispanic white, non-Hispanic black and Hispanics as three separate categories.

Survey design of the study was declared using SVYSET statement of STATA 13.1 [18]. Percentages and 95% Confidence Intervals (CI) were computed for all categorical variables of interest. Shapiro-Wilk test was used to test for normality of all continuous variables and shapes of the distributions were assessed using histograms. Since the continuous variables were non-normally distributed, medians and interquartile ranges were used for summary statistics. Chi-square tests were used to investigate the bivariate relationship between each potential predictor variable and the outcome of interest (diabetes status: diabetes, pre-diabetes and neither). Statistical inferences were based on a critical p≤0.05.

### Multinomial Logistic Model

The first step in building the multivariable multinomial logistic model involved fitting simple multinomial models between each of the potential predictors, and the polytomous diabetes status variable. Variables that were significantly associated with the outcome (p≤0.05) were considered for inclusion in the multivariable multinomial model. However, to avoid highly correlated predictor variables, two-way correlations between the predictor variables were assessed using Pearson’s correlation coefficient.

Manual backwards elimination procedure was used to fit a weighted (to account for the complex sampling design) multivariable multinomial model with all predictors that had simple associations; setting the p-value for removal at 0.05. Confounding was assessed using a change in parameter estimate of 20% [19] when the model is run with and without a specific suspected confounder of interest. Variables that either had a significant association (p≤0.05) with the outcome, or resulted in at least a 20% change of the parameter estimates of the variables already in the model were retained in the model to form the final main effects model. Age was forced in the model due to the apriori belief that it was a confounder. Two-way interaction terms of the variables in the final main effects model were then assessed for statistical significance. Significant ones were retained in the final model. Relative risk ratios (RRR) and their 95% confidence intervals (CI) were then computed for all variables in the final model.

Model goodness-of-fit was assessed using the goodness-of-fit test proposed by Fagerland, Hosmer, and Bofin using STATA’s estimation command mlogitgof [20]. Hosmer-Lemeshow goodness of fit test was also used to assess the fit of each of the ordinary logistic regression portions of the multinomial model as proposed by Dohoo, Martin and Stryhn [19]. The impact of individual observations on the model were assessed using graphical techniques. All statistical analyses were performed using Stata version 13.1 [18].

### Ethical Statement

This study was approved by the University of Tennessee, Knoxville Institutional Review Board. Since this was a retrospective study, informed written consent could not be obtained from the study participants. However, records of all participants were anonymized and de-identified before the study data were released to the investigators.

## Results

### Prevalence Estimates

This study included a total of 34,186 survey respondents, of which 2,983 (7.3%) and 5,189 (12.1%) had been told by a doctor that they had pre-diabetes and diabetes, respectively. Table 1 shows the characteristics of the respondents. The respondents were mostly women (52%), married (59.5%) and overweight (36.4%) individuals. Additionally, they were predominantly white (59.6%) and 31% had above high school education. Thirty-eight percent of the respondents reported earning ≥ $50,000 annually (Table 1). Moreover, most (77%) respondents reported having no health care coverage. The percentage of self-reported hypertension, hypercholesterolemia and arthritis were 35%, 40% and 26%, respectively. Most respondents reported consuming more than one fruit (62%) or vegetable (79%) per day. Both age and amount of sleep the respondents reported were markedly non-normally distributed (p < 0.0001). The age of the respondents ranged from 18 to 99 years, with a median of 61 and interquartile range of 48 to 72. Reported amount of sleep over a 24-hour period ranged from 1 to 24 hours, with a median of 7 and interquartile range of 6 to 8.

**Table 1:** Demographic, Health, and Lifestyle Characteristics of Adults 18 Years and Older in Florida Who Were Included in the Behavioral Risk Factor Surveillance Study, 2013*.

Characteristic	Categories	n (in 1000’s)	**Weighted % (95% CI)***
**Age category (years)**			
	18–24	1,377	11.54 (10.64, 12.50)
	25–34	2,518	15.48 (14.55, 16.46)
	35–44	3,196	15.38 (14.48, 16.33)
	45–54	5,149	17.59 (16.68, 18.53)
	55–64	7,331	16.51 (15.70, 17.36)
	65 or older	14,615	23.51 (22.74, 24.29)
**Sex**			
	Male	13,340	48.36 (47.16, 49.57)
	Female	20,846	51.64 (50.43, 52.84)
**Race**			
	White (non-Hispanic)	27,368	59.61 (58.40, 60.80)
	Black (non-Hispanic)	2,947	13.93 (13.01, 14.89)
	Other race (non-Hispanic)	1,353	4.31 (3.87, 4.79)
	Hispanic	2,518	22.16 (20.96, 23.41)
**Marital status**			
	Married	14,192	59.49 (58.21, 60.76)
	Never married	2,720	14.52 (13.49, 15.62)
	Separated/divorced/ widowed	10,851	25.99 (24.95, 27.06)
**BMI (kg/m** ^**2**^ **)**			
	Underweight (< 18.5)	720	2.27 (1.95, 2.64)
	Normal (18.5–24.9)	10,815	34.96 (33.79, 36.15)
	Overweight (25–29.9)	11,597	36.40 (35.20, 37.61)
	Obese (≥ 30)	9,420	26.38 (25.33, 27.44)
**Education**			
	< High school	3,394	14.96 (13.91, 16.08)
	High school	10,679	30.15 (29.03, 31.29)
	Some college	9,904	31.18 (30.11, 32.27)
	College	10,209	23.71 (22.85, 24.59)
**Income level**			
	< $15,000	4,222	14.40 (13.44, 15.43)
	$15,000–< $25,000	6,390	20.40 (19.35, 21.48)
	$25,000–< $35,000	3,798	12.44 (11.6, 13.33)
	$35,000–< $50,000	4,415	14.71 (13.85, 15.62)
	≥ $50,000	10,346	38.04 (36.83, 39.27)
**Healthcare coverage**			
	Yes	4,858	22.86 (21.78, 23.99)
	No	29,145	77.14 (76.01, 78.22)
**Diabetes status**			
	Pre-diabetes	2,983	7.31 (6.76, 7.90)
	Diabetes	5,189	12.06 (11.32, 12.85)
	No Pre-diabetes/Diabetes	24,707	80.63 (79.70, 81.53)
**Hypertension**			
	Yes	15,684	34.59 (33.50, 35.69)
	No	18,390	65.41 (64.31, 66.50)
**Hypercholesterolemia**			
	Yes	14,445	40.33 (39.12, 41.56)
	No	15,771	59.67 (58.44, 60.88)
**Arthritis**			
	Yes	13,242	26.02 (25.11, 26.94)
	No	20,655	73.98 (73.06, 74.89)
**Smoked** ≥ **100 cigarettes**			
	Yes	16,679	54.93 (53.74, 56.12)
	No	16,399	45.07 (43.88, 46.26)
**Consume vegetable(s)**			
	< 1 per day	5,824	20.83 (19.80, 21.90)
	≥ 1 per day	24,491	79.17 (78.10, 80.20)
**Consume fruit(s)**			
	< 1 per day	11,588	37.95 (36.73, 39.17)
	≥ 1 per day	19,390	62.05 (60.83, 63.27)
**Physical activity**			
	Highly Active	9,688	31.38 (30.18, 32.60)
	Active	4,152	15.72 (14.75, 16.73)
	Insufficiently Active	4,245	16.55 (15.57, 17.59)
	Inactive	10,112	36.35 (35.03, 37.70)

*Data Source: Behavioral Risk Factor Surveillance System

**Column subtotals may not sum to the total due to missing data,

***95% Confidence Intervals.

### Simple Associations

The following variables had significant simple associations with the polytomous diabetes status variable: age (p < 0.0001), sex (p = 0.0288), marital status (p < 0.0001), BMI (p < 0.0001), hypertension (p < 0.0001), hypercholesterolemia (p < 0.0001), arthritis (p < 0.0001), educational level (p < 0.0001), income level (p < 0.0001), having any health care coverage (p < 0.0001), smoking at least 100 cigarettes and physical activity (p < 0.0001) (Table 2). However, race (p = 0.0815), fruit intake (p = 0.8251) and vegetable consumption (p = 0.3277) were not significantly associated with diabetes status. Additionally, simple multinomial models indicated that older adults who were or had been married were more likely to be pre-diabetic and/or diabetic compared to the younger adults and those who had never been married. Obese individuals, those who reported that they had hypertension, hypercholesterolemia, and arthritis or had smoked at least 100 cigarettes were more likely to be pre-diabetic or diabetic. In addition, persons who were insufficiently active (Less than 150 minutes of moderate-intensity, or 75 minutes of vigorous-intensity physical activity per week) or inactive were more likely to be pre-diabetic and/or diabetic compared to those who were active. There was no statistically significant association between the amount of sleep and either pre-diabetes (p = 0.650) or diabetes (p = 0.468).

**Table 2:** Prevalence of Predictors and their Bivariate Relationship with the Polytomous Diabetes Status Variable among Adults 18 Years or Older in Florida, 2013*.

		Pre-diabetes	Diabetes	No Pre-diabetes / Diabetes	
		(n = 2,983)	(n = 5,189)	(n = 24,707)	
Characteristic	Categories	% (95% CI)	% (95% CI)	% (95% CI)	P-value
**Age (years)**					<0.0001
	18–24	0.32 (0.22, 0.49)	0.13 (0.06, 0.30)	10.85 (9.92, 11.84)	
	25–34	0.70 (0.48, 1.01)	0.34 (0.20, 0.58)	13.66 (12.75, 14.64)	
	35–44	1.09 (0.84, 1.41)	0.72 (0.54, 0.96)	12.68 (11.85, 13.57)	
	45–54	1.21 (1.01, 1.44)	2.04 (1.70, 2.44)	14.60 (13.73, 15.52)	
	55–64	1.59 (1.35, 1.87)	2.88 (2.52, 3.29)	12.54 (11.78, 13.34)	
	65 or older	2.40 (2.17, 2.66)	5.95 (5.46, 6.49)	16.30 (15.66, 16.95)	
**Sex**					0.0288
	Male	3.61 (3.19, 4.09)	6.41 (5.84, 7.03)	39.01 (37.78, 40.26)	
	Female	3.70 (3.36, 4.07)	5.65 (5.16, 6.19)	41.62 (40.41, 42.83)	
**Race**					0.0815
	White (nonHisp)	4.65 (4.31, 5.03)	7.24 (6.77, 7.74)	47.87 (46.73, 49.01)	
	Black (nonHisp)	1.15 (0.88,1.49)	1.81 (1.50, 2.19)	11.11 (10.24, 12.04)	
	Other race (nonHisp)	0.34 (0.24, 0.49)	0.45 (0.31, 0.65)	3.30 (2.92, 3.72)	
	Hispanic	1.17 (0.89, 1.53)	2.57 (2.11, 3.12)	18.35 (17.16, 19.61)	
**Marital status**					<0.0001
	Married	5.21 (4.71, 5.77)	8.45 (7.78, 9.17)	45.92 (44.61, 47.23)	
	Never married	0.91 (0.72, 1.14)	0.92 (0.74, 1.14)	12.76 (11.74, 13.86)	
	Sep./divor./ widow.	2.44 (2.15, 2.78)	4.76 (4.23, 5.35)	18.63 (17.75, 19.55)	
**BMI (kg/m** ^**2**^ **)**					<0.0001
	Underweight (< 18.5)	0.07 (0.04, 0.13)	0.10 (0.06, 0.18)	2.07 (1.74, 2.45)	
	Normal (18.5–24.9)	1.37 (1.14, 1.63)	1.73 (1.51, 2.00)	31.38 (30.20, 32.59)	
	Overweight (25–29.9)	2.74 (2.38, 3.16)	4.46 (3.95, 5.02)	29.60 (28.42, 30.81)	
	Obese (≥ 30)	3.12 (2.76, 3.51)	5.92 (5.38, 6.51)	17.45 (16.53, 18.41)	
**Education**					<0.0001
	< High school	0.92 (0.69, 1.21)	2.89 (2.40, 3.48)	11.52 (10.53, 12.60)	
	High school	2.22 (1.90, 2.59)	3.73 (3.37, 4.14)	24.57 (23.47, 25.71)	
	Some college	2.42 (2.12, 2.75)	3.37 (3.02, 3.77)	25.27 (24.24, 26.33)	
	College	1.76 (1.54, 2.00)	2.07 (1.84, 2.33)	19.26 (18.45, 20.09)	
**Income level**					<0.0001
	< $15,000	1.25 (0.95, 1.64)	2.56 (13.57, 15.64)	10.77 (9.89, 11.72)	
	$15,000–<$25,000	1.48 (1.20, 1.83)	3.00 (19.34, 21.53)	15.93 (14.94, 16.96)	
	$25,000–<$35,000	0.96 (0.79, 1.18)	1.70 (11.59, 13.37)	9.79 (8.99, 10.65)	
	$35,000–<$50,000	1.13 (0.92, 1.38)	1.79 (14.04, 15.89)	12.02 (11.19, 12.91)	
	≥ $50,000	2.56 (2.25,2.91)	3.08 (36.38, 38.88)	31.99 (30.79, 33.21)	
**Healthcare coverage**					<0.0001
	No	6.23 (5.73, 6.76)	10.56 (9.87, 11.29)	60.64 (59.41, 61.86)	
	Yes	1.09 (0.86, 1.38)	1.55 (1.25, 1.93)	19.93 (18.85, 21.05)	
**Hypertension**					<0.0001
	No	3.27 (2.86, 3.74)	3.04 (2.63, 3.51)	57.98 (56.78, 59.17)	
	Yes	4.05 (3.69, 4.44)	9.02 (8.38, 9.69)	22.64 (21.65, 23.67)	
**Hypercholesterolemia**					<0.0001
	No	3.40 (2.96, 3.90)	4.78 (4.19, 5.44)	50.99 (49.68, 52.31)	
	Yes	4.86 (4.41, 5.35)	9.24 (8.56, 9.96)	26.74 (25.64, 27.87)	
**Arthritis**					<0.0001
	No	4.43 (3.97, 4.93)	6.14 (5.56, 6.78)	62.89 (61.77, 63.99)	
	Yes	2.83 (2.53, 3.16)	5.94 (5.46, 6.46)	17.78 (17.01, 18.57)	
**Smoked** ≥ **100 cigarettes**					<0.0001
	No	3.61 (3.17, 4.11)	5.50 (4.97, 6.09)	45.40 (44.13, 46.67)	
	Yes	3.74 (3.40, 4.10)	6.65 (6.09, 7.26)	35.11 (33.95, 36.28)	
**Consume vegetable(s)**					0.3277
	< 1 per day	1.41 (1.15, 1.72)	2.77 (2.37, 3.24)	16.84 (15.85, 17.87)	
	≥ 1 per day	5.89 (5.40, 6.43)	9.41 (8.71, 10.15)	63.68 (62.43, 64.92)	
**Consume fruit(s)**					0.8251
	< 1 per day	2.911 (2.56, 3.31)	4.583 (4.12, 5.10)	30.37 (29.17, 31.59)	
	≥ 1 per day	4.527 (4.07, 5.04)	7.559 (6.91, 8.26)	50.05 (48.75, 51.36)	
**Physical activity**					<0.0001
	Highly Active	2.19 (1.91, 2.50)	3.34 (2.97, 3.77)	26.13 (24.95, 27.34)	
	Active	1.06 (0.87, 1.28)	1.84 (1.50, 2.26)	12.37 (11.45, 13.34)	
	Insufficiently Active**	1.26 (1.00, 1.58)	1.81 (1.53, 2.14)	12.96 (12.03, 13.96)	
	Inactive	3.06 (2.64, 3.53)	6.30 (5.64, 7.02)	27.71 (26.42, 29.03)	

*Data Source: Behavioral Risk Factor Surveillance System

**Insufficiently active = Less than 150 minutes of moderate-intensity, or 75 minutes of vigorous-intensity physical activity per week

***95% Confidence Intervals.

### Multivariable Multinomial Logistic Model

**Fig 2:**
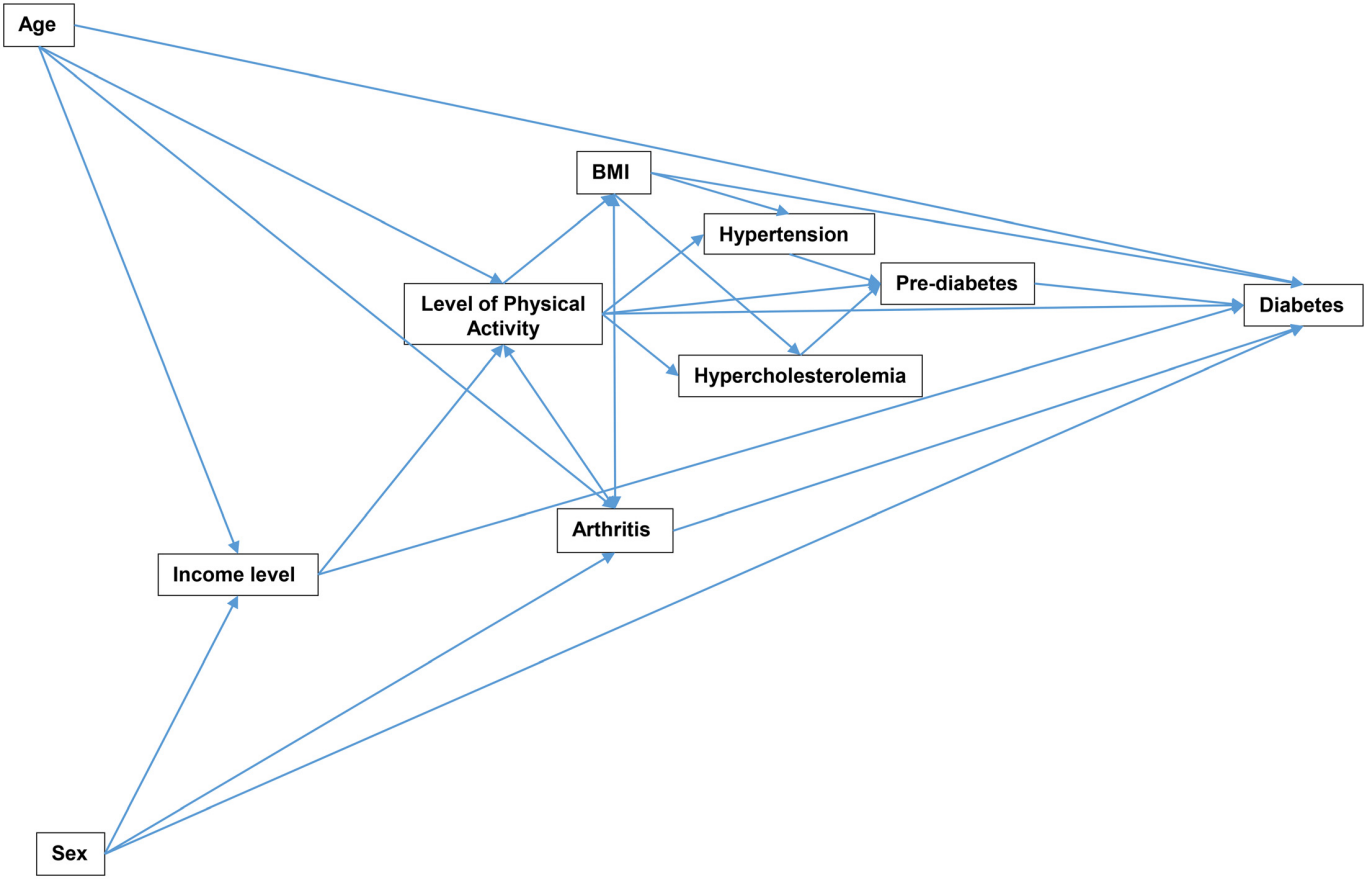
Conceptual Model Representing Only the Significant Predictors of Pre-diabetes and Diabetes Based on the Results of the Multinomial Logistic Model for Adults 18 Years and Older in Florida, 2013

Table 3 shows the results of the final multinomial logistic model. The conceptual model was revised based on the results of this model and significant predictors are presented in Fig 2. Based on this model, the following factors were associated with pre-diabetes: being overweight (Relative Risk Ratio (RRR) = 1.85, 95% CI: 1.41, 2.42) or obese (RRR = 3.41, 95% CI: 2.61, 4.45) or hypertensive (RRR = 1.69, 95% CI: 1.33, 2.15) or hypercholesterolemic (RRR = 1.94, 95% CI: 1.55, 2.43) as well as being arthritic (RRR = 1.24, 95% CI: 1.00, 1.55). The factors that had statistically significant association with pre-diabetes also had statistically significant associations with diabetes. However, stronger associations were observed for each of the predictors of diabetes compared to pre-diabetes. Moreover, some variables such as age, sex, income level and level of physical activity had significant associations with diabetes but not pre-diabetes.

**Table 3:** Final Multivariable Multinomial Logistic Regression Model Investigating Predictors of Pre-diabetes and Diabetes among Adults 18 Years or Older in Florida, 2013*.

		Pre-diabetes		Diabetes	
Characteristic	Categories	**RRR (95% CI)	P-value	RRR (95% CI)	P-value
**Age Group (years)**					
	65 or older	1.36 (0.64, 2.89)	0.42	13.33 (1.81, 98.14)	0.011
	55–64	1.21 (0.57, 2.56)	0.618	9.40 (1.28, 69.22)	0.028
	45–54	0.94 (0.45, 1.97)	0.866	5.86 (0.79, 43.44)	0.083
	35–44	1.06 (0.48, 2.33)	0.889	3.75 (0.49, 28.54)	0.202
	25–34	0.61 (0.27, 1.38)	0.239	2.47 (0.29, 20.91)	0.408
	18–24	Reference group			
**BMI**					
	Underweight (< 18.5)	0.56 (0.27, 1.13)	0.103	0.53 (0.28, 0.98)	0.044
	Normal (18.5–24.9)	Reference group		Reference group	
	Overweight (25–29.9)	1.85 (1.41, 2.42)	< 0.0001	2.00 (1.55, 2.57)	< 0.0001
	Obese (≥ 30)	3.41 (2.61, 4.45)	< 0.0001	4.04 (3.22, 5.07)	< 0.0001
**Hypertension**					
	Yes	1.69 (1.33, 2.15)	< 0.0001	2.66 (2.08, 3.41)	< 0.0001
	No	Reference group			
**Hypercholesterolemia**					
	Yes	1.94 (1.55 2.43)	< 0.0001	1.98 (1.61, 2.45)	< 0.0001
	No	Reference group			
**Arthritis**					
	Yes	1.24 (1.00, 1.55)	0.053	1.28 (1.04, 1.58)	0.022
	No	Reference group			
**Income level**					
	< $15,000	1.43 (0.91, 2.24)	0.123	2.40 (1.67, 3.43)	< 0.0001
	$15,000–<$25,000	1.14 (0.86, 1.52)	0.363	1.78 (1.36, 2.33)	< 0.0001
	$25,000–<$35,000	1.23 (0.91, 1.65)	0.18	1.76 (1.29, 2.40)	< 0.0001
	$35,000–<$50,000	1.15 (0.85, 1.57)	0.368	1.25 (0.95, 1.65)	0.106
	≥ $50,000	Reference group			
**Sex**					
	Male	1.08 (0.86, 1.35)	0.501	1.39 (1.12, 1.71)	0.002
	Female	Reference group			
**Level of physical activity**					
	Inactive	1.20 (0.93, 1.54)	0.157	1.59 (1.26, 2.00)	< 0.0001
	Insufficiently Active	1.00 (0.74, 1.37)	0.973	1.21 (0.92, 1.59)	0.169
	Active	0.99 (0.73, 1.35)	0.96	1.36 (1.00, 1.84)	0.051
	Highly Active	Reference group			

*Data Source: Behavioral Risk Factor Surveillance System

**RRR = relative risk ratio.

For instance, being 55–64 years (RRR = 9.40, CI: 1.28, 69.22), and 65 years or older (RRR = 13.33, CI: 1.81, 98.14) increased the risk of diabetes but not pre-diabetes. Furthermore, being male (RRR = 1.39, 95% CI: 1.12, 1.71), having an income of less than $15,000 (RRR = 2.40, 95% CI: 1.67, 3.43), $15,000–<$25,000 (RRR = 1.78, 95% CI: 11.36, 2.33), $25,000–<$35,000 (RRR = 1.76, 95% CI: 1.29, 2.40) and being physically inactive (RRR = 1.59, 95% CI: 1.26, 2.00) also significantly increased the risk of diabetes but not pre-diabetes. It was interesting to note that the risk of diabetes decreased with increasing income levels. No biologically meaningful significant interactions were detected.

## Discussion

This study was designed to estimate the burden of pre-diabetes and diabetes in Florida and to investigate their predictors in this population. Most previous studies have investigated the predictors of either pre-diabetes or diabetes in separate models. Thus, no past studies have investigated the two conditions in the same model in an attempt to assess how the associations between the outcomes (pre-diabetes and diabetes) and the suspected predictors vary depending on the level of the outcome. Therefore, this study is among the first to use multinomial models to investigate the predictors of both pre-diabetes and diabetes. Although multinomial logistic regression model offers insight into risk factors and their complex relationships with health outcomes, not many studies have used them. The modeling approach used in this study (i.e. multinomial logistic regression) provides an insightful tool to epidemiological investigations and is important in the investigation of categorical outcomes with more than two levels. The findings of this study will be important for guiding programming decisions and resource allocation for disease control and prevention.

Although the prevalence of pre-diabetes and diabetes continue to rise in the United States [21], it remains widely unreported at the state level compared to available documentation at the national level. This makes understanding existing disparities for both conditions challenging. A prevalence study in Florida reported 2010 estimates of self-reported diabetics to be 10.4% (95% confidence interval [95% CI]: 9.8, 11.1) [22]. Our study indicates the prevalence has since increased to 12.1% (95% CI: 11.32, 12.85). This finding is consistent with reports by the CDC illustrating an increasing trend in prevalence of both conditions over the past decade [13]. Furthermore, the results of our study show that the prevalence of pre-diabetes and diabetes increased with increasing age and BMI. Thus, the increase in pre-diabetes and diabetes can be closely linked to an increasing aging population, as well as, a worsening obesity problem.

Evidence from this data suggests that self-reported pre-diabetes and diabetes is significantly associated with being overweight or obese, hypertensive, hypercholesterolemic, and arthritic. Several studies have identified significant associations of pre-diabetes and diabetes with these risk factors among diverse populations [23–31]. However, to our knowledge, this is the first to identify a significant association between arthritis and pre-diabetes.

Some differences were observed in the degree of association for some of the predictor variables depending on the diabetes status. For instance, age was not significantly associated with pre-diabetes, whereas it was significantly associated with diabetes. The observed increasing odds of diabetes with age is consistent with findings from other studies [32–36]. The association between diabetes and increasing age is related to the increase in glycated hemoglobin levels and the changes in insulin sensitivity which is measured by the Quantitative Insulin Sensitivity Check Index (QUICKI) [33, 37, 38]. Thus, identifying older adults with pre-diabetes may be important to help initiate early preventive or treatment measures, thus decreasing the development to diabetes, thus decreasing its burden, and subsequently decreasing healthcare costs.

In contrast to findings from previous studies that found significant associations between pre-diabetes and sex [39], there was no significant association between pre-diabetes and sex, and the reason for this remains unclear. However, our findings suggest that females had lower risk of diabetes than males. This could be due to lower detection rates in women since they are more likely to have impaired glucose tolerance (IGT) without impaired fasting glucose (IFG) compared to men [24]. Moreover, women are more likely to undergo fasting glucose tests instead of OGTTs [24]. On the contrary, males are at higher risk of having impaired fasting glucose than females leading to higher pre-diabetes and thus diabetes in males than females [40, 41]. These sex differences may also be due to differences in body size, genetics and in fasting glucose levels as women have been reported to have overall better insulin sensitivity [24, 39].

Being inactive (less than 150 minutes a week of moderate-intensity, or 75 minutes of vigorous-intensity aerobic physical activity, or an equivalent combination of moderate and vigorous-intensity aerobic activity) approximately doubles the risk of diabetes compared to being highly active. Also, individuals who were just active (30 minutes a day of physical activity) had a higher risk of diabetes than those who were highly active. On the contrary, physical activity was not significantly associated with pre-diabetes after controlling for age and the other predictors in the final model. This is consistent with findings from a study that examined the relationship between physical activity and pre-diabetes in which subjects who were the most physically active were 0.77 times as likely to be pre-diabetic as their BMI matched controls who were not as physically active, but these effects were erased when controlled for age; even among those participants who achieved the recommended 30 min of daily moderate to vigorous physical activity [42]. Our findings on diabetes are comparable to other epidemiological studies that suggest that high level physical activity for more than 30 minutes a day or 150 minutes a week is significantly associated with a reduced risk of diabetes [25]. This is because high levels of physical activity aids the absorption of the hormone insulin into all the body’s cells, including the muscles, thus speeding up blood flow to the muscles and increasing energy consumption that translates to lower risk of diabetes by lowering blood glucose levels [43]. High levels of physical activity may also result in muscle building that enhances the body’s ability to utilize glucose better than fat. Therefore, building muscle can help prevent higher than normal blood glucose levels. Additionally, high levels of physical activity helps control body weight through increased fat metabolism which also significantly reduces the risk of diabetes [25, 44]. It is worth noting that the association between physical inactivity and diabetics with the trend of increasing BMI among pre-diabetes and diabetes in this and other studies, points to a very real area of focus for prevention programs [25, 45, 46].

Income level was used as one of the indicators of socioeconomic status (SES), and a potential predictor of pre-diabetes and diabetes. In this study, there was no significant association between pre-diabetes and income strata although other studies have shown that lower SES is generally expected to be associated with poor health outcomes [23, 47], However, significant associations were found between diabetes and income at the three lowest income levels (< $15,000, $15,000–<$25,000, and $25,000–≥ $35,000) with lower odds of diabetes being associated with increasing income. Low income level is associated with poverty which can cause a 2–3 fold increase in the odds of developing diabetes [48]. This is because living in poverty generally means less access to education, which culminates to fewer opportunities for jobs that pay well, and provide health insurance [49, 50]. Moreover, individuals who cannot afford insurance are less likely to seek care for diabetes and hence suffer complications from diabetes (such as heart disease and stroke, blindness, kidney failure, and lower-limb amputation) if undiagnosed or treated [2]. Even with universal health coverage, poverty still increases the incidence of type 2 diabetes and inequality of care for existing cases [51]. Living in poverty also means lack of access to resources such as adequate and healthy nutrition, safe walking and biking trails, and recreational or exercise facilities which increases a person’s risk for developing other risk factors (such as obesity) associated with diabetes [52]. Additionally, some studies have found that association between low income levels and diabetes incidence remains significant after adjusting for age, sex, health behaviors, and psychological distress [53]. As a result, it is important that intervention strategies integrate poverty as a major risk factor for diabetes and develop health policies to reduce socioeconomic disparities, in particular income inequities, along with individual-level risk factors in order to effectively prevent, manage and reduce the overall burden of diabetes.

An important strength of our study is the multinomial modeling approach within a conceptual framework in investigating how the association between the outcomes (pre-diabetes and diabetes) and suspected predictors vary at different levels of the outcome. However, the study also had some limitations including lack of availability of data for some important potential predictors of the diseases. For instance, we did not explore the associations between alcohol intake, family history of diabetes and consumption of certain foods (such as fast foods) with the outcome of interest. It was not possible to investigate if the survey respondents had been subjected to an OGGT test in order to be defined as pre-diabetic or diabetic. Neither could we separate type 1 from type 2 diabetes which may differ in pathogenesis. Moreover, we could not determine if diabetic patients received treatment so as to better understand associations found in this study. Furthermore, since the BRFSS data are self-reported, data collected are subject to recall bias leading to potential under or over-reporting. For example, height and weight information obtained from the respondents in order to calculate the BMI is likely to be misreported. Thus, the percentage of the respondents with higher BMI may be under-reported. However, other studies have reported that self-reported information on diabetes status and sociodemographic characteristics from the BRFSS have proven to be accurate [54, 55]. Moreover, self-reported level of physical activity in survey data have also been shown to have high accuracy [56]. Thus, the above limitations notwithstanding, the findings from this study provide useful information to both guide future studies as well as health planning and programming decisions.

## Conclusions

The findings of this study show significant demographic disparities in the risk of pre-diabetes and diabetes in the study area with the highest risk observed among overweight or obese, hypertensive, hypercholesterolemic and arthritic adults for both conditions. This study provides some evidence to the fact that, there may be differences in importance of significant predictors between pre-diabetes and diabetes. When studied alone, it is assumed that the factors are equally important. Lifestyle modification programs can help high risk individuals with pre-diabetes from becoming diabetic. Therefore, intervention and prevention of both diseases may be effective if concentrated in these areas.

Multinomial logistic regression models provide insight into the differences in associations between the predictor and health outcomes at different levels (categories). Therefore, multinomial regression models are a useful statistical method in investigating the associations between disease outcomes with precursors such as pre-diabetes in order to better understand the predictors at different levels of the disease. Thus, this approach should be part of the investigative methods used by epidemiologist as it would help provide a further insight into predictors at different stages of the disease process. However, further research needs to be conducted to improve model diagnostics of multinomial regression models.
